# Many accurate small-discriminatory feature subsets exist in microarray transcript data: biomarker discovery

**DOI:** 10.1186/1471-2105-6-97

**Published:** 2005-04-13

**Authors:** Leslie R Grate

**Affiliations:** 1 Life Sciences Division, Lawrence Berkeley National Laboratory, Berkeley CA 94720 USA

## Abstract

**Background:**

Molecular profiling generates abundance measurements for thousands of gene transcripts in biological samples such as normal and tumor tissues (data points). Given such two-class high-dimensional data, many methods have been proposed for classifying data points into one of the two classes. However, finding very small sets of features able to correctly classify the data is problematic as the fundamental mathematical proposition is hard. Existing methods can find "small" feature sets, but give no hint how close this is to the true minimum size. Without fundamental mathematical advances, finding true minimum-size sets will remain elusive, and more importantly for the microarray community there will be no methods for finding them.

**Results:**

We use the brute force approach of exhaustive search through all genes, gene pairs (and for some data sets gene triples). Each unique gene combination is analyzed with a few-parameter linear-hyperplane classification method looking for those combinations that form training error-free classifiers. All 10 published data sets studied are found to contain predictive small feature sets. Four contain thousands of gene pairs and 6 have single genes that perfectly discriminate.

**Conclusion:**

This technique discovered small sets of genes (3 or less) in published data that form accurate classifiers, yet were not reported in the prior publications. This could be a common characteristic of microarray data, thus making looking for them worth the computational cost. Such small gene sets could indicate biomarkers and portend simple medical diagnostic tests. We recommend checking for small gene sets routinely. We find 4 gene pairs and many gene triples in the large hepatocellular carcinoma (HCC, Liver cancer) data set of Chen *et al*. The key component of these is the "placental gene of unknown function", PLAC8. Our HMM modeling indicates PLAC8 might have a domain like part of lP59's crystal structure (a Non-Covalent Endonuclease lii-Dna Complex). The previously identified HCC biomarker gene, glypican 3 (GPC3), is part of an accurate gene triple involving MT1E and ARHE. We also find small gene sets that distinguish leukemia subtypes in the large pediatric acute lymphoblastic leukemia cancer set of Yeoh *et al*.

## Background

Transcriptional profiling studies can produce data in the form of abundance measurements for genes in samples assigned to one of two classes. A recent exemplar employed cDNA microarrays to assay 6605 clones from normal liver and liver cancer (hepatocellular carcinoma) tissues [1]. Given such two-class high-dimensional data, one analytical task is identifying a "small" subset of features able to discriminate between the classes. Tools that solve this problem would accelerate development of novel and/or improved molecular targets for diagnosis, prognosis, and therapy [2]. For example, enunciating genes able to distinguish liver cancer from normal samples could assist investigations into the etiology and treatment of liver cancer.

Existing classification and feature selection techniques can be employed to ascertain the cardinality of a feature subset yielding a classifier that generalizes well, *i.e*., one which makes zero (or few) errors in assigning the class of an unseen data point. Frequently, application of these approaches to a data set results in the definition of one discriminatory subset with tens to hundreds of features and requiring similar numbers of free parameters. This work focuses on subsets smaller than those produced by existing algorithms: all subsets of one-, two-, (and sometimes three-) features that can be separated by a linear surface without error. A multiplicity of error-free linear classifiers constructed from few features could facilitate the creation of cost-effective clinical tests and guide further basic research.

Here, an *m*-feature classifier is defined as a decision surface for *m*-dimensional data points where the *m *features are a subset of *P a priori *features, *m *≪ *P*. The potential number of these classifiers is equivalent to choosing m items out *P*, *i.e*., . This number increases when different types of decision boundaries are permissible for each value of *m*. The scope of the problem can be reduced and simplified if only *m*-feature *linear *classifiers (*m*-LCs) are considered. This restriction of neglecting non-linear decision surfaces is reasonable because hyperplanes can be calculated efficiently, and Support Vector Machines with linear kernels are sufficient for classification problems associated with profiling data (see for example [3-6]). Recent work by Bo [7] and Kim [8] demonstrate the utility of looking for small feature sets. Bo and Jonassen surveyed a number of classifier discovery methods including linear hyperplanes. They showed that accurate two-gene classifiers exist in real world data sets and that they perform well. They only analyzed 2 data sets, did not report computer runtimes nor consider single genes or gene triples in their analysis. Kim *et al *employed a heuristic, Monte Carlo-based strategy to discover 2- and 3-LCs for a real-world, 3226-dimensional, two-class transcriptional profiling data set [8]. This sophisticated method computes noise tolerant hyperplanes using an analytic spherical model. However, 140 hours on a supercomputer cluster were required to identify at least 11 pairs of genes, each of which separates the data. Thus, although brute force exhaustive search provides a comprehensive and systematic method for finding all small discriminatory feature subsets, the strategy is expensive computationally and largely untenable for *m *≥ 3 (the problem size grows combinatorially). The high dimensionality of transcriptional profiles and the logistical issues associated with exhaustive enumeration of all 1-, 2- and 3-LCs have lead to the prevailing assumption that such searches are both too expensive and unlikely to be informative. Thus, while some recent studies have made use of Kim's method [9-12], most profiling studies neither consider nor report small sized discriminatory feature subsets.

Here, a relatively inexpensive method for calculating maximal margin hyperplanes, LIKNON [6,13], is utilized to rapidly find error-free m-LCs in ten published transcriptional profiling data sets that assayed samples from liver, human breast, ovary, lung, skin, gastrointestinal tract, bone marrow, brain, and prostate. The number of free parameters in this method is *m *+ 1, hence is very small relative to the size of the data, greatly reducing the problem of over-fitting to the training data (see Random Data section). It seems plausible that the existence of single genes and gene pairs with the ability to form perfect linear classifiers may be a widespread phenomenon. To demonstrate the biological utility of the strategy, the gene pairs and triples discovered in the aforementioned LiverCancer data set were examined and found to yield new and unanticipated scientific insights. Overall, the results indicate the importance of ascertaining, as a matter of routine, the presence (or absence) of small distinguishing feature subsets.

## Results

All results are available through the web site [14].

The 10 published cancer data sets examined here are listed in Table 1. They range in size from small (few genes and/or a small class) to large (many genes with large classes), using a variety of microarray technologies. For each data set, all single and pairs of genes were tested (for some data sets gene triples were tested) using the LIKNON technique. All gene sets that formed zero training error classifiers were saved and are available via the web site. Table 2 lists the number of such gene sets. Many data sets have single genes or pairs that form such perfect linear classifiers which is interesting as most original reports did not note their presence. Computer runtimes are given in Table 3. A rough time estimate is 1 second per million pairs per sample. Evaluating all pairs requires checking about 0.5 * *n*^2 ^pairs (a triangular matrix). So a 2000 gene, 30 sample set would need about  seconds.

As expected, small data sets are found to have many thousands of gene pairs while large data sets have few. The gene sets discovered in the two large data sets (LiverCancer and YeohALL) are likely biologically relevant and are discussed later. The thousands of pairs found on small data sets are mostly due to the small sample size of the data, and would likely not maintain their perfect classification upon addition of further patient samples. Generalization performance was estimated with a LOO methodology (see Methods) and is often reasonably good.

However, even gene sets found in small data sets can be interesting depending on the end use. For use in a medical diagnostic it is desirable that the gene set be highly accurate on large number of patient samples so the test result error rate is low. If used to guide basic research, even a poor error rate could indicate a productive research direction.

**Figure 1:**
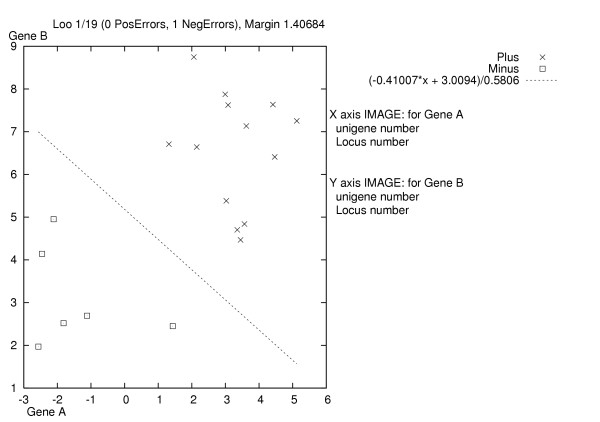
**Example plot using two hypothetical genes**. Each data point is labeled with the class, and the separating plane is computed to be positioned halfway between the two classes. In this example there is a large separation between the two classes and perfect separation is achieved and no data point is close to the plane.

The results are easily visualized for single and pairs of genes by a scatter plot. For each sample the expression values for the gene(s) set the x-y position and the point is labeled with the sample class as shown in the example plot Figure 1. The separating plane is drawn between the two clouds of points and one can see the amount of separation between the two classes (which is twice the "margin") a particular set of genes pair yields. A larger margin means the data are more well separated hence is more resilient to noise and more likely biologically relevant.

## Discussion

Many of the genes sets found accurately classify the data with large separation between the classes. It is exciting to consider the possibilities for medical diagnostics if some small gene set is found to accurately and reproducibly indicate a disease state. While general machine learning principles suggest that having more features (genes in this case) is desirable in order to make more noise resistant classifiers, this is data dependent and gene pairs with a training error rate of 1/90 as found in the LiverCancer data could be perfectly acceptable. Small gene sets, even if not accurate enough for medical purposes, can indicate fruitful new research directions. Like other classification methods that produce large numbers of genes, considering the corpus of all genes found can provide insight to the underlying biology.

Experimental design and construction of a data set profoundly influences the presence of small sized classifiers. For example, the BRCA sets are labeled according to their known BRCA1/BRCA2 mutation status. If some of the measured genes reflect this mutation status we would apriori expect to find some (possibly many) small feature sets, and not finding any could indicate errors in the class labels (a sample is mislabeled).

In data where the class sizes are small, most of the small gene sets will prove sensitive to noise. Thus they are not likely to perform well as predictors on new samples, or in different experiment setups. Classifiers made from large data sets are more likely to be reproducible and perform well in other situations. Classifiers with large margin and zero LOO error are more likely to indicate real biological effects that would hold true on new patient samples.

The GIST and BreastBRCA experiments are examples of both of the above conditions, and both lead to very large numbers of pairs. Both have small class sizes and have pre-disposed differences between the classes. The GIST experiment compares cancers from different tissue types which means there will be a very strong signal from just the tissue differences rather than just the cancers alone. The BreastBRCA experiment has the pre-disposition of being split along BRCA status lines. In both cases the number of patient samples and class sizes is small and 100,000+ pairs are found. We suspect there are some biologically real pairs hidden in the large background noise due to small sample sizes. Random data tests (see Methods) indicate that 30+ samples with more even class sizes are needed in order to reduce the random chance noise to a very small level (Table 4).

Gene sets that perform well across independent experimental data sets also likely indicate real biological effects. However, cross-experiment array comparisons are difficult and would be much easier and more broad if experimenters used more common clones and references.

Is the cost of such computation worth it? Certainly it is for single genes and pairs. Modern computers are powerful enough to solve these size problems in a few minutes. Triples needs tens to hundreds of hours on a single computer, which is still tractable. Quadruples are beyond single computer tractability. However, this type of algorithm is trivially parallelized over a standard network of computers leading to linear speed up. Each computer would be instructed to examine a given part of the search space and thereafter be independent from all the rest. A super computer or dedicated computer cluster is not required. It seems possible that the occurrence of such small sized classifiers is a common characteristic of microarray data, thus making the effort of searching for small gene sets worth the computational cost. The technique is not restricted to RNA/DNA transcript microarray data as used here. It can be applied to protein microarray, mass-spectra, or any data with similar characteristics.

### Data set discussion

We can't discuss all the gene sets found in all data sets: there are too many. Here we discuss results from the 2 large data sets that we think produce highly biologically relevant results. The supplement [see Additional file 1] contains further discussion and the web site [14] provides access to all the results and plots.

### Liver cancer

The large liver cancer data set contains 2 classes (tumor and normal) with 181 patient samples and measurements for 6605 genes. We examined this data set in more detail than the others as it is large and any results found are likely to be biologically relevant. The original data [1] was re-normalized using the Intensity/local then Spatial/local methods as implemented in the BioConductor R package [15] (this is the best performing method as outlined in, Wei Wu, unpublished 2004). With the original data example labelings (105 tumor, 76 normal), there are no pairs found. However, normal LIKNON [6] discovers a 23 gene classifier with 2 of the tumor examples strongly mis-classified (patient samples 108 and 109 in the raw data table from [1]). This suggests these 2 samples might have some sort of problem with them (contamination with too much normal tissue, a very different type of cancer, different tumor stage, etc) or are simply mislabeled. Relabeling these 2 tumor samples to be normal results in 4 gene pairs being found. Going further we wished to look for gene triples, yet using all 6605 genes would lead to excessive runtimes, so we applied a variation filter to reduce the number of genes. This variation filter (requiring a variation of at least 3.6 in *log*_2 _values for each gene) reduced the number of genes examined from 6605 down to 1956, which yielded 43 gene triples (using the original labelings) and 9496 gene triples (with the 2 "outlier" genes relabeled). Only 291 of these triples were saved for further analysis. All 4 gene pairs, 35 of the 43 and 229 of the 291 triples include the recently annotated gene PLAC8 (IMAGE:491644) a "placenta specific gene of unknown function".

The 4 genes in pairs with PLAC8 are

• IMAGE:669379 GLCCI1 glucocorticoid induced transcript 1

• IMAGE:590591 ADCY6 adenylate cyclase 6

• IMAGE:260259 Transcribed sequence with moderate similarity to protein sp:P39188 (H.sapiens)

• IMAGE:756490 BCAT2 branched chain aminotransferase 2, mitochondrial

**Figure 2:**
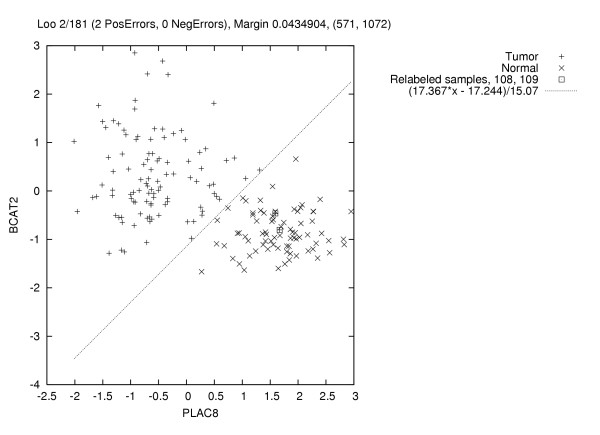
**Liver cancer pair PLAC8 verses BCAT2**. The two misclassified samples 108 and 109 are shown as squares. There are 3 other genes that form such pairs with PLAC8.

Figure 2 shows the plot of the pair PLAC8 and BCAT2. There is good separation between the tumor and normal samples, except for the 2 "outlier" examples. Most of the triples found when the "outlier" examples are relabeled have larger margins than those found with the original data labels. The top triple with the original labeling is

**Figure 3:**
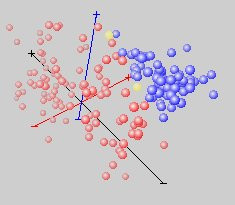
**Liver cancer 3D plot of MT1E, ARHE and GPC3**. These 3 genes form a perfect classifier although the margin is small. Red are cancer samples. The web site contains an interactive plot.

• IMAGE:1472735 MT1E metallothionein 1E (functional) Hs74170

• IMAGE:784593 ARHE ras homolog gene family, member E Hs6838

• IMAGE:878564 GPC3 glypican 3 Hs119651

and is shown in Figure 3. GPC3 is a recently noted HCC cancer marker [16], where it was elevated in 6 out of 7 patient samples. Here this gene triple makes no errors on all 181 patient samples. The top triple using the relabeled examples is

• IMAGE:78353 RNAHP RNA helicase-related protein Hs8765

• IMAGE:491644 PLAC8

• IMAGE:667883 PHLDA1 pleckstrin homology-like domain, family A, member 1 Hs82101

In all, the 291 triples make use of 168 of the genes. The fact that triples exist when using the original labelings argues that the "outlier" examples are correctly labeled as tumor, albeit maybe a different type of tumor (or in a different development stage). That these small gene sets exist in such a large data set argues that they are biologically relevant. These 4 gene pairs only make 2 errors out of the 181 patient samples (the two "outlier" samples are the errors), which is an error rate of 2/181 = l/90. The triples found with the original labelings make no errors. Based on such data, one can imagine a simple few-gene diagnostic test based on these pairs and triples.

It is perhaps not surprising that a placental gene is associated with liver cancer. Both are blood organs, and cancers often recapitulate early development stages, of which the fast growing placenta might be an example. In addition, mitochondria related genes have been associated with cancer progression.

For this data, normal LIKNON was a useful aid in identifying outlying data examples. The two outlying tumor samples in this data set could represent a rare tumor type or development state. Using such aids *during *the experiments would allow such samples to be identified in a timely manner for further investigation.

#### Modeling of PLAC8

**Figure 4:**
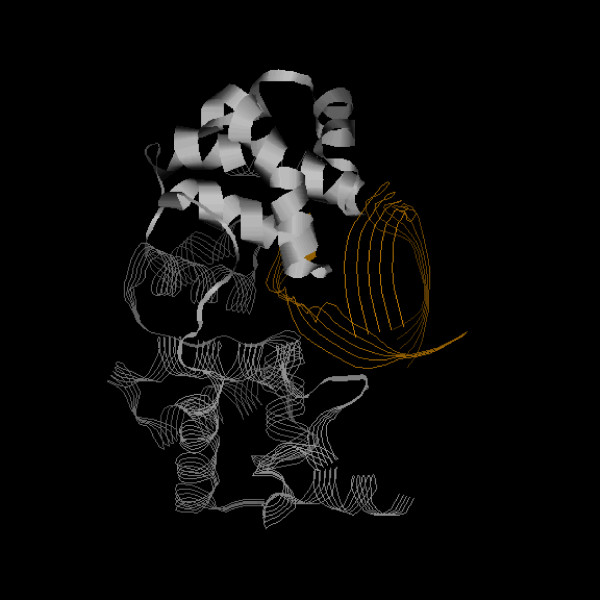
**1P59 crystal structure**. Shown with the alignment hit to the liver cancer possible biomarker PLAC8 highlighted in strands at the top. Alignment generated from PFAM model *pfam*04749.5*DUF*614 using the SAM HMM system and displayed in RASMOL.

PLAC8 is noted to have a match to the PFAM model pfam04749.5 DUF614. A search of the PDB database using a SAM HMM model [17] created from the PFAM alignment finds a hit to 1P59 (gi|34811270|pdb|1P59|A) which is a Non-Covalent Endonuclease Iii-Dna Complex from *bacillus stearothermophilus*. The PFAM model locates to the C terminus of the 1P59 crystal structure. The sequence of 1P59 is some 85 amino acids longer than PLAC8, and the alignment hit aligns the last 110 amino acids or so. Visualizing the 3D structure of 1P59 with the alignment hit in PLAC8 colored silver in the RASMOL tool (Figure 4), shows that this hit forms a distinct mostly helical domain at the C terminus.

### YeohALL

The YeohALL data set is a large multi-class pediatric acute lymphoblastic leukemia cancer set. The original data contains 7 classes, we use only 6 of them (we do not use their "other" class). For each of the 6 classes we compare each class against all others combined, thus asking the question can each class be distinguished from all the others. There are 248 patient samples and the 6 classes are T, E2A, BCR, TEL, MLL and Hyperdiploid > 50. Both the T vs the rest and E2A vs the rest splittings have single genes that perfectly separate T or E2A from the others. Five out of the 6 splittings have gene pairs, only Hyperdiploid vs the rest does not have any small gene sets. We applied a variation filter to reduce the original 12625 Affymetrix probes down to 4196 genes (the filter level used requires a variation of at least 10000 within each gene). We only discuss the top results for the T and E2A splittings here, see the supplement [see Additional file 1] for more details. Our results reinforce many of the findings in Yeoh's work that there are single genes that accurately classify the T and E2A leukemia subtypes and extends it by identifying accurate 2 gene classifiers.

#### T vs the rest

**Figure 5:**
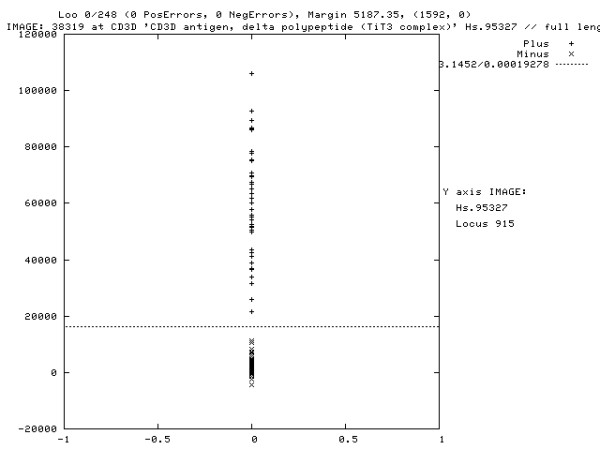
**From the YeohALL data, T vs the rest, the best single gene CD3D**. This gene perfectly separates the classes. Plus signs are T subtype samples.

**Figure 6:**
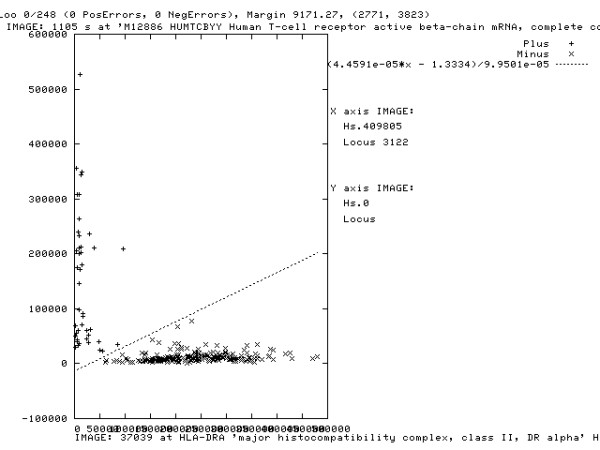
**From the YeohALL data, T vs the rest, the best pair HLA-DRA and HUMTCBYY**. Each gene alone provides some classification power, but when linearly combined form a perfect classifier, albeit with a small margin. Plus signs are the T subtype samples.

The only single gene separating T vs the rest is the same one identified by Yeoh, "38319_at CD3D antigen, delta polypeptide (TiT3 complex) Hs95327". This gene has a high value in T and a low value otherwise (Figure 5) and clearly separates the data. There are 1169 pairs making use of 681 different genes, the top pair is "37039_at HLA-DRA major histocompatibility complex, class II, DR alpha Hs409805", and "1105_s_at M12886 HUMTCBYY Human T-cell receptor active beta-chain mRNA" (Figure 6). In T, HUMTCBYY is generally high and HLA-DRA is low. These and many other of the genes in the top pairs are identified by Yeoh as being significantly differentially expressed in T. However they did not identify any gene pairs as accurate classifiers.

#### E2A vs the rest

The 4 single probe sets for E2A are

**Figure 7:**
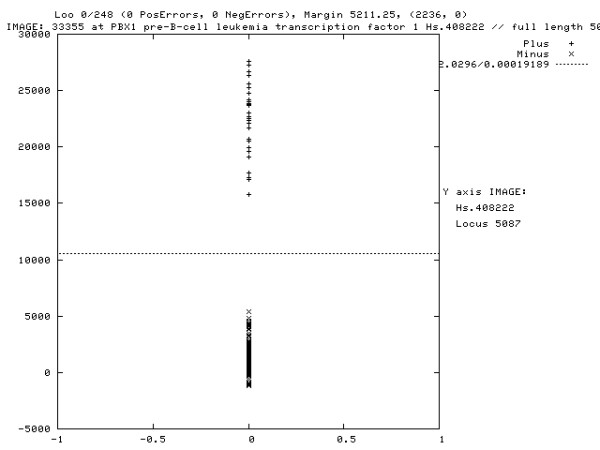
**From the YeohALL data, E2A vs the rest, the best single gene PBX1**. This gene perfectly separates the classes with a wide margin and has higher values in E2A. Plus signs are E2A subtype samples.

• "33355_at PBX1 pre-B-cell leukemia transcription factor 1 Hs408222"

• "1287_at ADPRT ADP-ribosyltransferase (NAD+; poly (ADP-ribose) polymerase) Hs177766"

• "430_at NP nucleoside phosphorylase Hs75514"

• "32063_at PBX1 pre-B-cell leukemia transcription factor 1 Hs408222"

(note that there are two probes for PBX1 both giving the same result, so there are really only 3 genes) All of these are high in E2A and lower in the rest. Probe 33355_at for PBX1 is shown in Figure 7 where it clearly separates the classes and was the only single gene identified in Yeoh's original work. The other 3 probes barely separate the data and likely failed Yeoh's stringent cross-validation criteria. These 4 probes are in Yeoh's significantly differentially expressed list.

**Figure 8:**
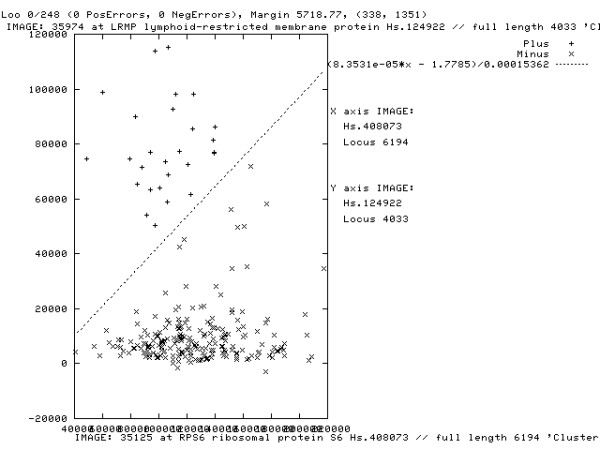
**From the YeohALL data, E2A vs the rest, the best gene pair RPS6 and LRMP**. Plus signs are the E2A subtype samples. LRMP by itself is a reasonable indicator of E2A status, but when combined with RPS6 can perfectly separate the data.

There are 386 pairs, the best pair is "35125_at RPS6 ribosomal protein S6 Hs408073" and "35974_at LRMP lymphoid-restricted membrane protein Hs124922". Figure 8 shows that this pair separates the data well. LRMP is identified in Yeoh's lists (but not RPS6), as are many of the genes in the other top pairs. These and the other top pairs often highly separate the data indicating they might be biologically relevant and resilient to noise. These and all the rest of the results are available through the web site.

## Conclusion

Small sets of genes (single genes, pairs of genes, and triples of genes) able to accurately classify two-class microarray data occur in many real-world data sets. These small sets could portend simple medical diagnostics and point to important research targets. The many small sized gene sets found here were not noted previously in the literature, and seemingly went unnoticed. Many members of the pairs discovered here have known associations with cancer and indicate the possibility of simple, accurate medical diagnostic tests based on such results.

Exhaustively examining all pairs in thousand gene size datasets is easily tractable on modern computer hardware. All triples is harder, needing a few days, but this is only computer time, and powerful computers are cheap and plentiful. Given that the compute time is small enough and the results possibly important, we conclude that examining microarray data for single genes, gene pairs and maybe gene triples should be done routinely. When performed along with acquiring the biological data, the results can be used as a quality check on the experimental process.

The gene of unknown function, PLAC8 appears to have a role in Liver cancer, and based on our HMM modeling might have a domain similar to part of the crystal structure of 1P59. We find that there are 4 genes that when paired with PLAC8 form a classifier with the low error rate of 1/90. In addition there are many gene triples, often including PLAC8, that form zero error classifiers and might be good biomarkers. We find the previously identified HCC biomarker gene, glypican 3 (GPC3), is part of an accurate gene triple involving MT1E and ARHE. We also find small gene sets able to accurately distinguish leukemia subtypes in the large pediatric acute lymphoblastic leukemia cancer set of Yeoh *et al*.

## Methods

### Transcriptional profiling data sets

The existing transcriptional profiling data sets investigated here are summarized in Table 1. We generally chose to minimally process the data. cDNA microarray data were *log *transformed; Affymetrix data were used as is. Only the LiverCancer data set was subjected to advanced re-normalization procedures. The one data set with a few missing values (LungStanford) had them set to the appropriate class average value. If there were too many genes (operationally defined as more than about 5000), a variation filter was used to remove genes that didn't vary enough across all samples. The filterings (if used) were applied once, before LIKNON analysis, solely to reduce the number of genes and hence the runtimes. They were not used to adjust for "good" results. When three or more categories of samples had been defined by the original authors, two-class data sets were produced by partitioning these categories. Table 1 provides statistics for the final data matrices used as input to LIKNON for determining *m*-LCs.

### *m*-feature linear classifier (*m*-LC)

Consider a two-class data set composed of *N *data points, . Each data point is a *P*-dimensional vector of features, **x**_*n *_∈ ℝ^*P*^, assigned to one of two classes, *y*_*n *_∈ {+1, -1}. Assume that the classes are linearly separable. A classifier for such data is a hyperplane parameterized by a weight vector, **w **∈ ℝ^*p*^, and an offset from the origin, *b *∈ ℝ. A hyperplane, (**w**, *b*), can be used to predict the class of a data point **x **∈ ℝ^*P *^by computing sign(**w**^*T*^**x **+ *b*). If this value is positive, **x **is identified with the +1 class, otherwise it belongs to the -1 class. Data points that define the hyperplane, positive half-space (+1 class), and negative half-space (-1 class) are the sets {**x**|**w**^*T*^**x **= *b*}, {**x**|**w**^*T*^**x **>*b*}, and {**x**|**w**^*T*^**x **<*b*} respectively. For two-class profiling data, an error free *m*-LC is a maximal margin hyperplane based on *m *of these *P *features, which assigns the class of every data point correctly. The number of free parameters in such models is *m *+ 1, so in this setting is quite low (2–4), which is much smaller than the number of samples (for the smallest data sets is 14, on the largest data this is 248). By optimizing of the choice of *m *this might be over fitting the data, but in the Random Data section we show that we are finding many more features than chance alone would account for.

Given data points specified by *P *features, the potential number of *m*-LCs is equivalent to the combinatorial problem of choosing *m *items out of *P*, *i.e*., . In this work all single genes (*m *= 1) and pairs (*m *= 2) and in some cases triples (*m *= 3) were evaluated using a linear sparse hyperplane method that has been described elsewhere (LIKNON) [6]. LIKNON determines a maximal margin hyperplane (for example in Figure 1) that separates the data classes. Maximal margin means that the hyperplane is positioned halfway between the two classes. Gene sets that linearly separate the data are recorded, otherwise they are rejected. If a single gene is a classifier, it is not used during the pair checks as it would always form a classifier with any other gene.

### Acceptance, generalization and error performance

This work accepts only perfect (no training error) linear hyperplane classifiers from the above method. This criteria for accepting only perfect classification is very stringent. We first thought that this would lead to few result sets being found, and that allowing non-perfect classification would need be done to find more classifiers. But this loosening turned out not to be necessary.

These models have only 2–4 free parameters. Thus the problem of over-fitting the model to the data during training is not a large issue and we don't perform any stringent generalization tests such as multi-round cross validation. Others interested in evaluating particular gene sets should perform such tests.

Generalization performance of classifiers was evaluated using a Leave-One-Out procedure. In LOO testing, one data point is removed and a classifier re-learned using the rest of the data. The resultant classifier is tested on the held out data point and if it is in error, a LOO error is counted. However for these *m*-LC's where *m *is small, LOO error is not a good performance measure. In general, only somewhat isolated points lying close to the classifier decision boundary will be found in error during LOO testing. Thus LOO error rates tend to be very low, 0 – 3 out of the total number of points. The small magnitude of this error count allows one to be misled thinking the classifier is "good because the LOO error is low". The LOO error is included in the results available from the web site.

In the end, classifiers with a larger separation (margin) between the classes are able to tolerate more noise without errors. Thus larger margin classifiers are more desirable for use on future data points.

### Implementation

The program is an adaptation of the lp_solve version of LIKNON [6] implemented in C. A modern desktop workstation (1.8 Ghz Athlon) is able to evaluate many thousands of pairs per second using this LIKNON method. Table 3 lists run times for some of the data sets. Evaluating all pairs requires about *n*^2^/2 (equivalent to filling in a triangular matrix), and all triples about *n*^3^/6. A data set with *P = *3000 features necessitates the evaluation of 4.5 × 10^6 ^pairs and 4.5 × 10^9 ^triples. An approximate average time is 1 second/10^6 ^pairs/sample. Thus, evaluating all pairs for these data sizes requires only a few minutes whereas all triples needs many hours.

### Random data

How many such pairs occur by chance? Theory suggests [18] that the probability of a 2 class data set of *N *items with *a *in one class, being linearly separable in 1 dimension is  and in 2 dimensions is approximately . Experiments were performed where a data matrix containing random numbers from a uniform distribution was analyzed by LIKNON. The results are shown in Table 4. When the number of samples is lower than about 30, or one class is very small relative to the other class, then the chance of finding a pair of random genes that form a perfect classifier is large enough to easily measure. The smallest real data set examined in this work (the GIST set) has 1987 genes in 19 samples with 13 in one class and 6 in the other. Experiments with 10 random data sets of this size shows that 1500 pairs would be expected on average with a maximum found of 2706, and no single genes, Table 5. Also in Table 5 are results of experiments where the real data and class labels are used, but the class labels are randomly shuffled. The label shuffling results are worse than random data only for the smallest two data sets (GIST and BreastBRCA BRCA1 vs BRCA2). The real GIST data has more than 137000 pairs, some 50 times more than found in random data and 30 times more than when labels are randomly shuffled. It also has 74 single genes, where the random data yields none. The three result sets that are closest to these random results are Cutaneous (596 vs 62), BRCA Breast BRCA1 & BRCA2 verses Sporadic splitting (2114 vs 1286) and BRCA Breast BRCA1 verses BRCA2 (143574 vs 53900).

## Web site

The web site [14] contains the data and results for this work.

## Authors' contributions

LG carried out all work outlined in this article. LG wrote and approved the manuscript.

## Supplementary Material

Additional File 1 The expanded discussion section. A discussion of the top results for all data sets. Click here for file

## Figures and Tables

**Table 1:** Published transcriptional profiling data sets reexamined in this study. For each set, the Table gives the abbreviation; dimensionality of the data points investigated after pre-processing of the features assayed in the original study; number, description and abbreviation for samples assigned to a category.

Name	Description
BreastER	Node-negative breast carcinomas, *P *= 3398 (3,398 cDNA clones) [19] 23 Estrogen receptor *α *positive (ER^+^) 24 Estrogen receptor *α *negative (ER-)
BreastBRCA	Primary breast tumors, *P *= 3226 (6,512 cDNA clones) [20] 7 BRCA1 mutation (BRCA1) 8 BRCA2 mutation (BRCA2) 7 Sporadic (Sporadic)
OvarianBRCA	Primary epithelial ovarian adenocarcinomas; *P *= 6445 (7,651 cDNA clones) [21] 18 BRCA1 mutation (BRCA1) 16 BRCA2 mutation (BRCA2) 27 Sporadic (Sporadic)
LungStanford	Lung tumors; *P *= 918 (blanks set to average, 24,000 cDNA clones) [22] 41 Adenocarcinomas (AC) 16 Squamous cell carcinomas (SCC)
LungBeer	Lung tissue samples; *P *= 4966 (4,966 cDNA clones) [23] 10 Non-neoplastic (Normal) 86 Adenocarcinomas (AC)
Cutaneous	Cutaneous melanomas; *P *= 3613 (the "detected" set, 8,150 cDNA clones) [24] 31 Melanoma biopsies (Melanoma) 7 Tumor cell lines (Cell line)
GIST	Tumors; *P *= 1987 (1,987 cDNA clones) [25] 13 *KIT*-mutation positive gastrointestinal stromal tumors (*KIT*^+^) 6 Spindle cell tumors from locations outside gastrointestinal tract (Spindle)
YeohALL	Pediatric, acute lymphoblastic leukemia bone marrows; *P *= 4196 (10000 variation filter, 12,625 Affymetrix HG_U95Av2 probes) [26] 43 T-lineage ALL (T) 27 E2A-PBX1 (E2A) 15 BCR-ABL (BCR) 79 TEL-AML1 (TEL) 20 MLL (MLL) 64 Hyperdiploid>50 (Hyperdip50)
Prostate	Prostate tissue samples; *P *= 3958 (12,626 Affymetrix U95a probes) [27] 25 Primary cancer tissue (Tumor) 9 Non-neoplastic tissue (Normal)
Liver	Liver Cancer (Hepatocellular carcinoma); *P *= 6605 (cDNA clones) [1] 105 Tumor (Tumor) 76 Normal (Normal)

**Table 2:** Number of 1-, 2- and 3-LCs for the data sets described in Table 1. For the two-class partitionings shown, the Table gives the number of perfect linear classifiers (given *P *initial features) that can be constructed using one, two and for some sets 3, genes. The 3 gene results report the total number of triples, and in parenthesis the number saved for later analysis.

Data set	Class +1	Class -1	1-LCs	2-LCs	3-LC
BreastER	ER^+^	ER^-^	0	16	108 k(2045/180^1^)
BreastBRCA	BRCA1	BRCA2	18	143,574	
	BRCA1/BRCA2	Sporadic	0	2,114	
	BRCA1	BRCA2/Sporadic	0	12,729	
	BRCA1	Sporadic	4	66,754	
	BRCA2	BRCA1/Sporadic	0	10,027	
	BRCA2	Sporadic	7	78,901	
OvarianBRCA	BRCA1	BRCA2	0	1,612	
	BRCA1/BRCA2	Sporadic	0	0	
	BRCA1	BRCA2/Sporadic	0	0	
	BRCA1	Sporadic	0	0	249^2^
	BRCA2	BRCA1/Sporadic	0	0	
	BRCA2	Sporadic	0	0	2^3^
LungStanford	AC	SCC	2	484	565 k(65)
LungBeer	AC	Normal	5	22,102	
Cutaneous	Melanoma	Cell line	0	596	4.2 m(386)
GIST	*KIT*^+^	Spindle	74	137,981	
YeohALL	T	E2A/BCR/TEL/MLL/Hdip50	1	1169	
	E2A	T/BCR/TEL/MLL/Hdip50	4	386	
	BCR	T/E2A/TEL/MLL/Hdip50	0	1	
	TEL	T/E2A/BCR/MLL/Hdip50	0	3	
	MLL	T/E2A/BCR/TEL/Hdip50	0	2	
	Hdip50	T/E2A/BCR/TEL/MLL	0	0	
Prostate	Tumor	Normal	52	249,665	
Liver	Tumor	Normal, Original Labels	0	0	44^4^
	Tumor	Normal, Relabeled	0	4	9.5 k(291^4^)

(^1^Only 180 of the estrogen triples do not contain the Estrogen Receptor 1 gene. ^2^Ovarian BRCA B1 vs Sporadic triples were run with a reduced set of genes, variation filtered to 2109. ^3^Ovarian BRCA B2 vs Sporadic triples were run with a reduced set of genes, variation filtered to 2097. ^4^Liver Cancer triples use only 1956 genes. Relabeled means the the 2 "outlier" samples are re-labeled as normal.)

**Table 3:** Wallclock run times for 2-LCs (pairs) for some of the data sets listed in Table. The *P*-LC program is written in C and uses double precision arithmetic. "Total time" (seconds) and "Time estimator" (seconds per 10^6 ^evaluations per sample) are execution times for the software on a 1.8 GHz AMD Athlon computer running Linux in an unloaded network configuration. An average time estimate is roughly 1 second per 10^6 ^evaluations per sample.

Data set	Number samples	Number genes	Number pairs	Total time	Time estimator
BreastER	47	3,389	5,700,000	251	0.94
BreastBRCA	22	3,226	5,200,000	161	1.41
LungBeer	96	4,966	12,300,000	1,500	1.27
Cutaneous	38	3,613	6,500,000	260	1.05
YeohALL	248	4,169	8,700,000	1,800	0.83

**Table 4:** Random data tests. The experimentally determined largest number of pairs found from 20 runs on random data. Each class has half the number of samples (number positives = number of negatives). The total number of genes is 2000. The second column is the number of pairs found for this 2000 gene set size, third column is the observed probability a single pair will be a perfect classifier ("observed" / 2000^2^/2).

Samples	observed	Chance for a single pair
16	4625	0.002312
18	1822	0.000911
20	965	0.0004825
22	510	0.000255
24	30	0.000015
26	10	0.000005
28	8	0.000004
30	1	0.0000005
32	1	0.0000005

**Table 5:** Random data simulations of real data sets. This table compares the results found from the real data (Real column) to two different types of random data. The Random column contains the experimentally determined largest number of pairs found from 10 simulation runs using a random data matrix (drawn from a uniform distribution) where the number of genes and class sizes is the same as the indicated for the real data. The Label Shuffled column contains the experimentally determined largest number of pairs found from 30 simulation runs where the class labels were randomly shuffled. In the samples column, the number in parenthesis is the number of positive samples. The numbers after the slash are the number of single genes found. Label shuffling leads to more pairs found "by chance" only for the smaller data sets. The small data sets have large numbers of pairs expected "by chance".

Data set	Samples	Genes	Real	Random	Label Shuffled
GIST	19(6)	1987	137981/74	2706/0	4622/2
BreastBRCA(brca1 vs brca2)	15(7)	3226	143574/18	20563/2	53900/11
BreastBRCA(brca1 & brca2 vs Sporadic)	22(7)	3226	2114/0	1286/1	0/0
Cutaneous	38(7)	3613	596/0	62/0	24/0
LungStanford	52(13)	918	486/2	0/0	0/0
LungBeer	96(10)	4966	22102/5	0/0	0/0
Prostate	34(9)	3958	249662/52	57/0	13/0
